# MEFV and NLRP3 Inflammasome Expression Is Attributed to Immature Macrophages and Correlates with Serum Inflammatory Proteins in Crohn´s Disease Patients

**DOI:** 10.1007/s10753-022-01647-8

**Published:** 2022-02-21

**Authors:** Frida Gorreja, Charles Caër, Stephen T. A. Rush, Sophia K. Forsskål, Anetta Härtlova, Maria K. Magnusson, Elinor Bexe Lindskog, Lars G. Börjesson, Mattias Block, Mary Jo Wick

**Affiliations:** 1 Department of Microbiology and Immunology, Institute of Biomedicine, University of Gothenburg, Box 435, 40530 Gothenburg, Sweden; 2 Biometrics, R&D, AstraZeneca, Mölndal, Sweden; 3 Wallenberg Centre for Molecular and Translational Medicine, University of Gothenburg, Gothenburg, Sweden; 4 Department of Surgery, Institute of Clinical Sciences, Colorectal Unit, Sahlgrenska University Hospital, Gothenburg, Sweden; 5 Inflammation Research Center (CRI), INSERM-UMR1149, University of Paris, Paris, France

**Keywords:** intestinal inflammation, epithelium, NLRPs, IL-6, TRAIL.

## Abstract

Inflammasomes are intracellular protein complexes whose activation results in proinflammatory cytokines. Inflammasomes are implicated in Crohn´s disease (CD) pathogenesis, yet the contribution of inflammasomes in intestinal epithelial cells (IECs) versus lamina propria (LP) macrophages is poorly understood. Whether inflammasome expression in intestinal tissue reflects the serum inflammatory protein profile of patients is also not known. We aimed to determine the intestinal cell types where inflammasome expression is increased in CD and if they correlate with the serum protein profile. RT-PCR and NanoString nCounter technology were used to characterize inflammasome gene expression in CD patients and controls. The mucosa, LP and IEC cell fractions and FACS-sorted cells were analyzed. Proximity extension assay with a 92-protein panel was used to determine the serum inflammatory protein profile. Compositional analysis was used to correlate ileum inflammasome gene expression with intestinal mononuclear phagocyte populations. We show that *NLRP3* and *MEFV* inflammasome sensors and downstream effector expression including *IL-1β* are increased in inflamed mucosa of IBD patients and correlate with disease activity. Inflammasome gene expression increased with the abundance of immature intestinal macrophages, and increased IL-1β released by CD LP cells correlated with immature macrophage frequency. Inflammasome gene expression was also increased in circulating monocytes, the precursors of immature intestinal macrophages. Finally, the serum inflammatory profile of CD patients correlates with ileal expression of genes related to NLRP3 and MEFV inflammasomes. Overall, we show that *MEFV* and *NLRP3* inflammasome expression in CD intestine is attributed to the accumulation of immature macrophages and correlates with serum inflammatory proteins.

**Supplementary Information:**

The online version contains supplementary material available at 10.1007/s10753-022-01647-8.

## Introduction

The inflammatory bowel diseases (IBD) Crohn’s disease (CD) and ulcerative colitis (UC) are chronic, immunologically mediated disorders of the gastrointestinal tract. Disease progression is a consequence of inflammation deriving from aberrant immune reactivity to commensal microbes, with genetics and environmental factors playing a role [1]. The chronic inflammation can lead to complications such as strictures, and it increases the risk of developing colorectal cancer (CRC), factors that force patients to undergo surgery [2]. The inflammation underlying IBD has a significant innate immune component, and intestinal cells including mononuclear phagocyte (MNP) subsets are involved in driving the inflammation [3]. MNPs are localized in the lamina propria (LP) underlying the epithelial layer of the gut mucosa and include conventional dendritic cells (cDCs), monocyte-derived immature macrophages (Mfs), and mature resident Mfs [3]. The dual role of intestinal Mfs in maintaining homeostasis and responding to pathogenic bacteria [3] underscores their pivotal role in the homeostasis/inflammation balance. Recent investigations of MNPs in the human intestine are sheding light into their function, and, although much remains to understand, aberrant activation of Mfs appears central to driving the inflammation of IBD [3, 4].

Treatments for IBD start with nonspecific anti-inflammatory drugs and typically escalate into biological treatment(s) targeting immunological pathways [2]. Biological treatments are widely used clinically but fail in a significant number of patients. Thus, additional treatments are needed. One possibility is dampening production of pro-inflammatory cytokines such as those in the IL-1 family [2]. Cytokines in this family, particularly IL-1β and IL-18, are produced as biologically inactive pro-forms that become active after cleavage by inflammatory caspases. Caspases themselves are harnessed as inactive enzymes within protein complexes called inflammasomes [5]. Inflammasomes are present in several cell types including intestinal epithelial cells (IECs) and MNPs. Inflammasomes are comprised of a sensor, caspases, and, in most cases, the adaptor ASC. Sensors give the name to the inflammasome and can be, for instance, microbial pattern-recognition receptors such as Nod-like receptors (NLRs), or other sensors such as Mediterranean fever (MEFV)/Pyrin.

Generating active IL-1β and IL-18 begins with assembly of the inflammasome complex in the cell cytosol. Assembly is initiated when the sensor is triggered by microbial products, such as LPS or peptidoglycan, and/or cellular damage or metabolic imbalance molecules. The assembled complex then activates caspases, which in turn cleave pro-IL-1β and pro-IL-18 into secreted active cytokines [5]. These cytokines, particularly IL-1β, act in combination with other pro-inflammatory cytokines, such as IL-6 and TNF, to drive IBD inflammation [6].

The most studied inflammasome in IBD is NLRP3, whose activation plays a role in the development of colitis in humans and IL-10^−/−^ mice [7]. Other inflammasomes studied in the context of IBD include MEFV/Pyrin, NLRP6, and AIM2 [8–10], which play a role in intestinal barrier integrity and inflammation [9, 10]. Despite an emerging link between inflammasomes and IBD, our understanding of the contribution of specific inflammasomes and intestinal cell types to inflammasome-generated cytokines in human disease is limited. This underscores the need to further our understanding of inflammasomes that govern the maturation of IL-1β and IL-18 in studies using patient material.

In an approach using mucosal tissue, LP and IEC cell fractions as well as FACS-sorted cells, we define transcriptional changes in inflammasome genes in the ileum and colon of IBD patients. We show for the first time a subset-specific contribution of the recently defined intestinal Mf subsets (Mf1-Mf4) to expression of inflammasome genes and IL-1β release. Finally, we show expression of key inflammasome-related genes correlates with disease activity and readily obtainable systemic measures, particularly inflammatory proteins differentially expressed in the serum of CD patients. These data further our understanding of the role of inflammasomes in CD and could facilitate development of new therapies to halt the inflammation that plagues IBD patients.

## Materials and Methods

### Study Samples

The demographics of study subjects is summarized in Tables 1 and 2. Tissue from CD and UC patients (Table 1) was obtained from patients undergoing surgery at the Department of Surgery, Sahlgrenska University Östra Hospital, Gothenburg, Sweden. CD cohort 1 was collected first (2011–2014) and consisted of mucosal tissue. CD cohort 2 was collected 2018–2020 and was used to confirm the findings from cohort 1. For CD cohort 2, we collected blood in addition to mucosal tissue at the time of surgery, and we refined cell preparation to isolate IECs and LP cells. Samples from UC patients were collected 2011–2014. All tissue from IBD patients was taken from unambiguously macroscopically inflamed sites as judged by experienced surgical IBD research nurses and endoscopists.

**Table 1:** Demographic Summary of the IBD Patients

	**CD cohort 1**	**CD cohort 2**	**UC cohort**
**Number of patients**	17	17	9
**Male/female**	3/14	10/7	8/1
**Age**^**1**^	41 (25–79)	47 (17–74)	34 (24–58)
**Disease duration (years)**^**1**^	13 (1–38)	10 (0–39)	11 (3–31)
**Harvey-Bradshaw Index**^**1**^	9 (3–24)	7 (0–20)	NA^2^
**Mayo Score**^**1**^	NA	NA	4 (3–9)
**Disease location**^**3**^ **(ileum/ileocolonic**^**4**^**/colon)**	2/9/6	9/8/0	0/0/9
**Blood (serum/PBMCs) taken**	0	11/9	0
**Treatments**			
**Corticosteroids**	10	14	7
**5-Aminosalicylic acid**	3	3	7
**Thiopurines**	8	6	4
**Anti-TNF**	9	8	3

^1^Data are shown as median (range)

^2^NA, not applicable

^3^Macroscopically inflamed tissue was taken from all patients

^4^Only inflamed ileum was taken from all patients with ileocolonic disease except for one patient in CD Cohort 1 where inflamed ileum and inflamed colon were taken

**Table 2:** Demographic Summary of the Control Subjects

	**Controls for CD cohort 1 and UC cohort (tissue)**	**Controls for CD cohort 2 (tissue)**	**Controls for CD cohort 2 (serum/PBMCs)**
**Number of subjects**	17	13	19
**Male/female/ND**	7/9/1	7/6/0	8/8/3
**Age**^**1**^	66 (22–94)	75 (61–86)	38 (25–74)
**Examination (endoscopy**^**2**^**/surgery**^**3**^)	5/12	0/13	NA^4^
**Type of sample (tissue/blood**^**5**^)	17/0	13/0	0/19
**Tissue collected** **(ileum/ileum and colon/colon)**	7/1/9	13/0/0	NA
**Samples prepared from blood (serum**^**6**^**/PBMCs)**	NA	NA	8/11

^1^Data are shown as median (range)

^2^Individuals undergoing endoscopy for health screening

^3^CRC patients undergoing surgery where macroscopically normal tissue at least 10 cm from the tumor was taken

^4^NA, not applicable

^5^Blood donors

^6^Serum was from age- and sex-matched blood donors

**Fig. 1:**
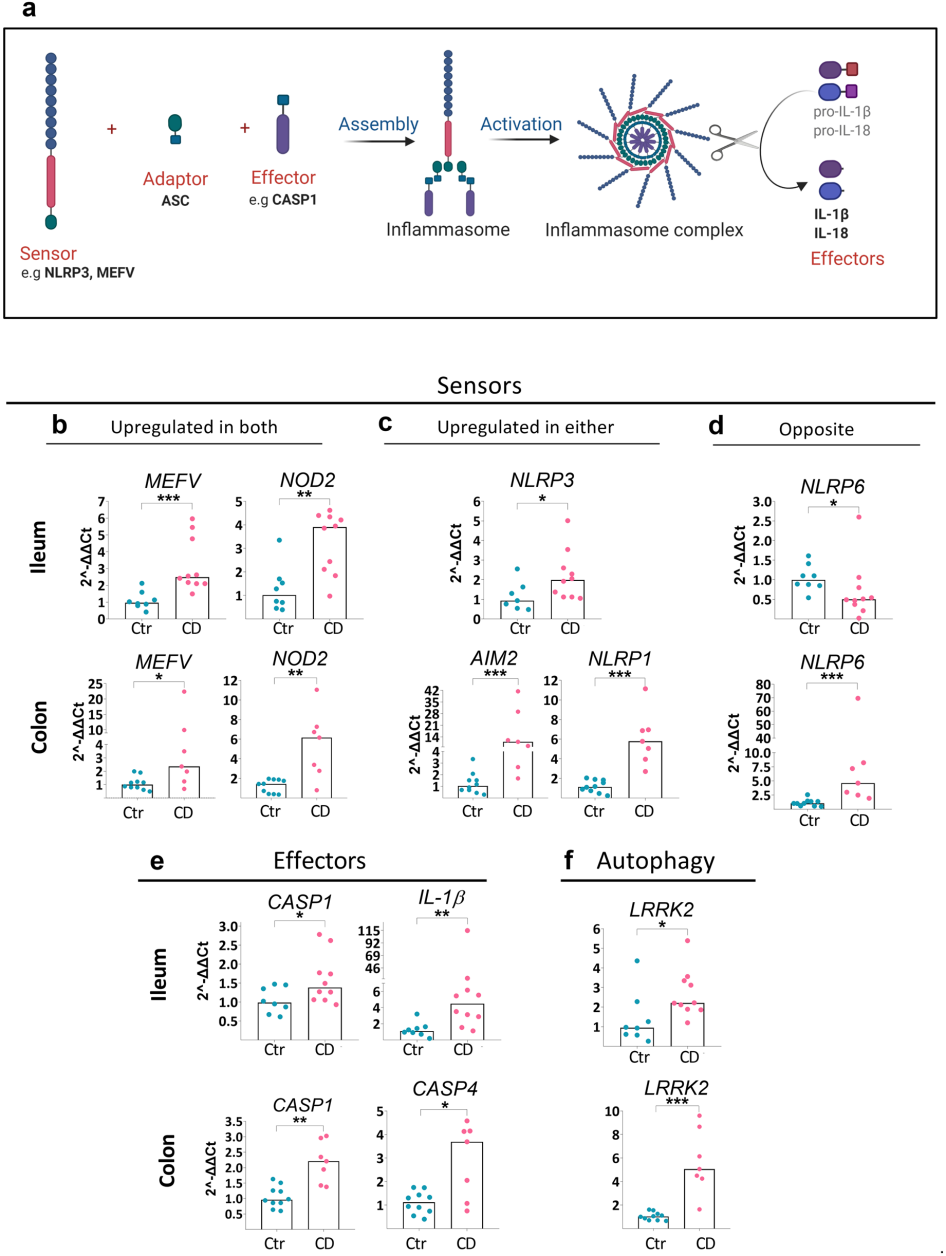
Gene expression of inflammasome components in intestinal mucosa of CD patients. Ileum from CD ileitis patients or colon from CD colitis patients (CD Cohort 1) with matching ileal/colonic control samples (Controls for CD cohort 1) were analyzed by RT-PCR. Gene expression was normalized to the housekeeping gene *RPLP0* and data are shown as relative fold change to control (Ctr) using the 2^−ΔΔCT^ method. (**a**) Schematic representation of components forming an inflammasome. (**b**–**d**) Gene expression of significantly differentially expressed sensors, effectors, and LRRK2, for either ileum (upper rows) or colon (lower rows) among the genes examined (Online Resource Table S1) is shown. Bar height shows the median. Ctr ileum *n* = 8; CD ileum *n* = 10; Ctr colon *n* = 10; CD colon *n* = 7. Significance was assessed by Mann–Whitney U test, *p < 0.05, **p < 0.005, ***p < 0.0005.

Control subjects are summarized in Table 2. Non-inflamed control tissue for the CD cohorts and the UC cohort was from patients undergoing tumor resection for CRC or endoscopy for health screening as indicated in Table 2. For CRC patients, macroscopically normal tissue at least 10 cm from the tumor was used. Likewise, macroscopically non-inflamed ileal tissue was taken from the five endoscopy controls for CD cohort 1 (Table 2) and used in Fig. 1. These biopsies were from individuals undergoing endoscopy for health screening that were recruited at the Endoscopy unit, Sahlgrenska University Hospital, Gothenburg, Sweden. Serum and PBMCs used as controls for CD cohort 2 (Table 2) were from anonymous healthy donors, and serum was from age- and sex-matched individuals.

### Primary Cell Isolation

IECs and LP cells were isolated as previously described [4, 11, 12]. Briefly, to obtain IECs, intestinal tissue was cut into pieces, incubated with 6–15 mL of pre-warmed HBSS without Ca/Mg (Gibco, UK) containing 2-mM EDTA (VWR, USA) for 15 min at 37 °C with stirring at 200 rpm. Supernatants were collected, fresh HBSS/EDTA was added to the tissue, and incubations were repeated 4–6 times. Supernatants were filtered on a 150-μm filter (SAATI, Italy) to remove debris and cells were collected. The 4–6 fractions of IECs were pooled and pelleted. Cells were washed with PBS and filtered (100 μm; Corning®Cell, Corning, USA), centrifuged, and incubated in 10–15 mL of red blood cell lysis buffer (154 mM NH_4_Cl, 10 mM NaHCO_3_, 0.1 mM EDTA) for 10–15 min at RT. Cells were counted (Glasstic-Slide10, KOVA), and viability was assessed by trypan blue exclusion and was routinely ~ 80%. Aliquots for flow cytometry and cell culture (to obtain conditioned media as described below) were removed and the rest was lysed for RNA isolation using RLT buffer plus 1% β-mercaptoethanol (RNeasy Plus Mini Kit, QIAGEN, Sigma-Aldrich, Germany).

To obtain LP cells, the remainder of the tissue from the EDTA digestion was incubated with 7–20 mL of RPMI 1640 containing GlutaMAX™-I (both from Gibco), 0.1-mg/mL Liberase DL (Roche, Germany), 60 Kunitz units/mL DNAse I (Sigma-Aldrich), and 2.5-mM CaCl_2_, 10% FBS (Biological Industries) at 37 °C stirring at 200 rpm for max 1.5 h. This digested tissue was dissociated using GentleMACS Dissociator (Miltenyi, Sweden). The resulting cell suspension was filtered using a 150 μm then a 40 μm filter (Corning), centrifuged and resuspended in red blood cell lysis buffer, and incubated for 10–15 min at RT. Cells were then washed and either lysed with RLT buffer, stained for flow cytometry or cultured to obtain conditioned media as described below.

### RT-PCR and nCounter Gene Expression

Mucosal samples were stored in RNAlater (Invitrogen) at − 80 °C until RNA extraction. RNeasy Plus Mini kit and TissueLyser II (both from Qiagen) were used to extract RNA for CD cohort 2, while NucleoSpin RNA kit (Macherey–Nagel GmbH, Belgium) was used for CD cohort 1 and the UC cohort. RNeasy Plus Mini Kit (Qiagen) was used for RNA extraction from IECs, LP cells, and PBMCs, while RNeasy Plus Micro Kit (Qiagen) was used for FACS-sorted cells. RNA concentration was measured using NanoDrop Spectrophotometer (ThermoFisher, USA). cDNA was synthetized from 500 ng of RNA using QuantiTect Reverse Transcription Kit (Qiagen). RT-PCR was done using QuantiFast SYBR Green PCR kit (Qiagen) on the 7500 Real Time PCR System (ThermoFisher). Transcriptional analysis of mucosa, IECs, and LP cells examined the genes shown in Online Resource Table S1a, while five genes were examined in PBMCs. Gene expression was normalized to the housekeeping gene *RPLP0*. All samples were run in at least duplicates.

For nCounter gene expression (NanoString, USA), RNA extraction was performed on 10-µm sections of formaldehyde-fixed paraffin-embedded tissue using the RNeasy FFPE kit (Qiagen). RNA quality was measured using TapeStation (Agilent 2200, USA), and, according to NanoString recommendations, preparations where at least 50% of the sample was greater than 300 nucleotides were sent for analysis. Gene expression was analyzed using the nCounter Fibrosis Panel (NanoString) plus 30 additional genes (Online Resource Table S1b). mRNA counts were normalized by NanoString to a pool of housekeeping genes.

### Proximity Extension Assay

Blood was collected into VacuetteZ Serum Sep Clot Activator tubes (Hettich, Sweden), processed within 3–4 h, and serum was stored at − 80 °C. Randomized samples were analyzed in one batch using the 92 protein Inflammation Panel (Olink, Sweden). Protein concentrations expressed as Normalized Protein Expression (NPX) (log_2_ arbitrary units) were used for statistical analysis. A limit of detection (LOD) was calculated by Olink via a negative control mimicking serum. Thirteen of the 92 proteins were below the LOD for > 50% of the samples. Proteins across patients with values below the LOD were used as such, as they represent the best estimate.

### Flow Cytometry and Cell Sorting

1 × 10^5^ − 5 × 10^6^ isolated cells were washed in FACS-buffer [PBS containing 3% FBS, 5 mM EDTA and 15 mM HEPES (Gibco)], incubated for 10 min in Live/Dead Fixable Aqua Dead Cell Stain Kit (Thermo Fisher) and binding inhibitor (eBioscience Human FcR) (ThermoFisher) in the dark at RT. LP cells were washed, incubated for 30 min with an antibody cocktail containing anti-EpCAM-FITC (Biolegend), anti-CD45-APC-H7 (BD Biosciences), three lineage exclusion markers (CD3, CD19, CD56; all PE-CF594 from BD Biosciences), HLA-DR-AlexaFluor®700 (Biolegend), CD14-BUV395 (BD Biosciences), CD11c-V450 (BD Biosciences), and CD11b-BUV737 (BD Biosciences). For IECs, cells were stained only with anti-EpCAM-FITC (Biolegend) and anti-CD45-APC-H7 (BD Biosciences). Flow cytometry was performed using either a LSR Fortessa X-20 W or FACSAria Fusion and analyzed using FlowJo (all from BD Biosciences). Flow cytometry data were used for FACS-adjusted gene expression analysis, compositional analysis, correlations, or cell sorting where indicated. For cell sorting, viable cells were gated, and IECs and LP cells were sorted as EpCAM^+^, CD45^+^, CD14^+^HLA-DR^−^, or EpCAM^−^CD45^−^ (Online Resource Fig. S1) using a FACSAria Fusion and used for gene expression analysis. To this end, 1 × 10^5^ − 1 × 10^6^ cells were sorted on low speed using a 100-μm nozzle into microcentrifuge tubes containing 500 μL of cold FACS-buffer, centrifuged and lysed with RLT buffer. The purity of each sorted cell type was ≥ 95%. Before RNA extraction, the same sorted cell type from at least four patients was pooled to obtain enough material for RT-PCR.

### Conditioned Media

IECs and LP cells were cultured at 1 × 10^6^ cells/mL in 96-well plates (NunclonDelta Surface, Thermo Fisher, Denmark) containing RPMI 1640 with 10% FBS and 0.1% gentamicin (Gibco) for 22 h at 37 °C. Conditioned media (CM) were centrifuged at 2000 rpm for 5 min and filtered (0.20 μm, Corning), generating IEC conditioned media (IEC-CM) or LP conditioned media (LP-CM). CM were stored with Protease Inhibitor Cocktail (Promega, USA) at − 80 ºC until used for IL-1β quantification.

### PBMC Isolation

PBMCs were obtained by density gradient separation (Ficoll-Paque PLUS, GE Healthcare Biosciences, Sweden) according to the manufacturer’s instructions. The separated PBMCs were incubated for 10–15 min with 5–10 mL of red blood cell lysis buffer, washed in PBS, and counted and lysed for RNA extraction using RLT lysis buffer plus 1% β-mercaptoethanol (RNeasy Plus Mini Kit-Qiagen, Sigma-Aldrich, Germany).

### FACS-Adjusted Gene Expression Analysis and Compositional Analysis

Differential gene expression in IECs and LP cells was assessed as follows. Cell fractions were split for dual use where one aliquot was used for flow cytometry analysis, and the remainder was used for RT-PCR. In the flow cytometry aliquots, the percent of EpCAM^+^CD45^−^, CD45^+^, and EpCAM^−^CD45^−^ cells was measured in both IECs and LP cells, whereas immature Mfs (CD14^+^CD11c^+^), mature Mfs (CD14^+^CD11c^−^), and cDCs (CD14^−^CD11c^−^) were analyzed only among LP cells (Online Resource Fig. S1). Cell percentage was used to generate FACS-adjusted gene expression data. This adjustment was done for EpCAM^+^, CD45^+^, EpCAM^−^CD45^−^ cell composition, or CD14^+^CD11c^+^, CD14^+^CD11c^−^ CD14^−^CD11c^−^ cells where indicated. FACS-adjusted gene expression data uses FACS as a compositional covariate and to adjust for cell composition in the models, the log-ratios of the cell abundances are used as covariates [13–15]. Differential gene expression was then assessed using ANCOVA while adjusting for the cell composition. Correlations were tested via Pearson correlations. The ternary plots were obtained after compositional analysis and display gene expression by cell composition of MNPs (CD14^+^CD11c^+^ immature Mfs, CD14^+^CD11c^−^ mature macrophages and CD14^−^CD11c^−^ cDCs).

### Biostatistics

R program 3.6.0 [16] (Packages: R-compositions, R-emmeans, R-ggtern, R-ppcor, R-ggplot2, R-ggbiplot R-lme4), GraphPad Prism 8 and FlowJo v10 were used for data analysis. Differential gene expression in mucosa and PBMCs was tested with the Mann–Whitney U test. For serum proteins, differential expression between controls and CD patients was modeled by a mixed effects model using log-NPX expression adjusted for age- and sex-matching stratum as a random effect. Hence, least-squares difference of means was tested. q value estimation was used for false discovery rate. PCA clustering was plotted in R. STRING was used to generate protein–protein interaction networks and associated pathways [17]. Pearson correlation was used to evaluate association between serum protein expression and FACS-adjusted IEC and LP gene expression. Given the exploratory nature of the study, power calculations were not performed. *p*​ values < 0.05 were considered statistically significant.

## Results

### Inflammasome Gene Expression Is Increased in Inflamed Mucosa of IBD Patients and Correlates with Disease Activity

To explore expression of inflammasome sensors and downstream effectors (Fig. 1a) in the ileum and colon of IBD patients, we performed transcriptional analysis of inflammasome-related genes on mucosal samples from CD patients undergoing surgery (CD cohort 1). Several sensors were significantly upregulated in inflamed mucosa of patients with CD compared to control tissue. This included *MEFV* and *NOD2* in both ileum and colon of CD (Fig. 1b), while others were upregulated in either (Fig. 1c). There was an opposite expression pattern for the sensor *NLRP6,* where it was reduced in CD ileum but increased in CD colon (Fig. 1d). Inflammasome effectors, such as *CASP1,* were upregulated in both the ileum and colon while *IL-1*β was upregulated in ileum (Fig. 1e). Finally, the autophagy-related gene *LRRK2* [18], which is susceptibility locus for CD and influences inflammation in colitis models, was upregulated in both CD ileum and colon (Fig. 1f). Thus, inflammasome genes are differentially expressed in intestinal mucosa of CD patients with differences between the ileum and colon.

**Fig. 2:**
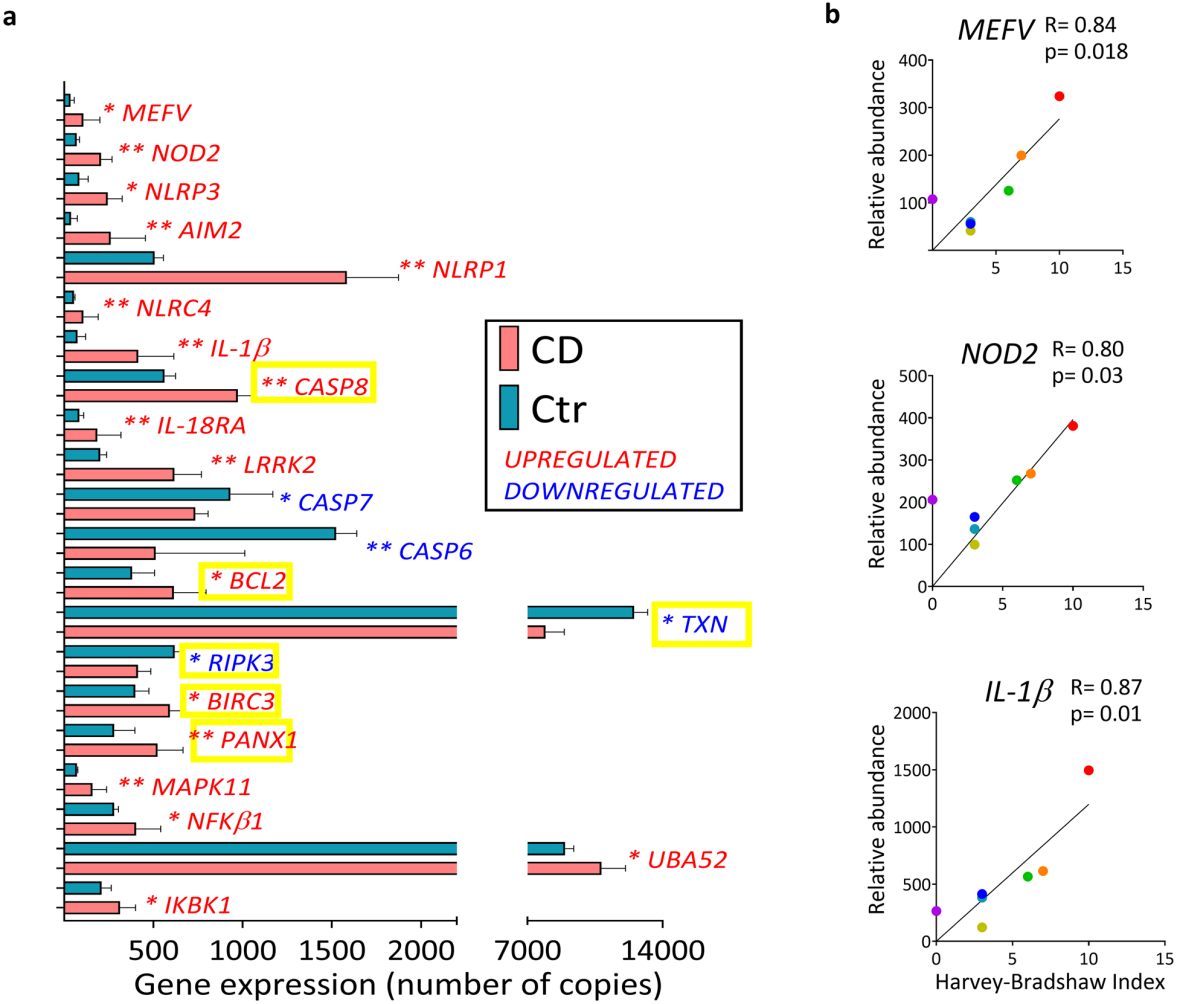
NLRP3-related genes are broadly activated in CD ileitis. Gene expression in ileal samples from CD cohort 2 and Controls for CD cohort 2 (Ctr) were analyzed using quantitative NanoString nCounter technology. Expression was normalized to the housekeeping gene pool and positive controls (by nCounter technology). (**a**) Expression of differentially expressed inflammasome-related genes in ileum is shown. Significance was assessed by Mann–Whitney U test, *p < 0.05, **p < 0.005. Data are shown as median IQR. NLRP3-related genes are indicated with a yellow box. (**b**) Linear regression plots show Pearson correlation of the indicated genes’ relative abundance with disease activity (Harvey Bradshaw Index). Ctr *n* = 5, CD *n* = 7.

We further addressed inflammasome expression and extended our analysis using quantitative NanoString nCounter technology and a panel containing 41 inflammasome-related genes [19]. Moreover, given that 80% of CD patients have ileal inflammation [2] analyses henceforth focus on ileitis patients (CD cohort 2) together with a new control cohort (Control cohort 2). nCounter technology revealed 21 differentially expressed inflammasome-related genes in CD ileum compared to control ileum (Fig. 2a). This included several genes that were upregulated in CD ileitis patients of both cohorts, while others were detected only in cohort 2 (Fig. 1b-f top rows and Fig. 2a). For example, *IL-1*β was upregulated in both cohorts, while differential expression of the effector *CASP1* in cohort 1 was not detected in cohort 2. Interestingly, nCounter analysis revealed altered expression of six genes downstream of NLRP3 inflammasome activation in CD ileum (Fig. 2a; yellow boxes). This suggests that signaling via the NLRP3 inflammasome may be increased in CD ileitis. Thus, several inflammasome genes are differentially expressed in ileal mucosa of CD patients, particularly in the NLRP3 pathway. Moreover, gene expression of several sensors, as well as downstream effectors including *IL-1*β, also correlated to the disease activity for CD patients with ileitis (Fig. 2b).

Finally, we addressed whether inflammasome gene expression is also altered in UC patients where, unlike CD, inflammation is restricted to the colon. In UC colon, expression of six inflammasome sensors increased relative to control where five were the same sensors that increased in CD colon (Online Resource Fig. S2a). Effectors *CASP1* and *CASP4,* and the autophagy-related gene *LRRK2*, were also increased in both CD colon and UC colon (Online Resource Fig. S2b-c and Fig. 1e-f). Notably, the cytokines *IL-*β and *IL-*18 were differentially expressed in CD colon and UC colon relative to controls. That is, *IL-1*β was increased in UC colon, but not in CD colon, while *IL-18* was reduced in UC colon but unchanged in CD colon (Online Resource Figs. S2b and 1e). Moreover, several inflammasome-related genes significantly correlated to the disease score of UC patients (Online Resource Fig. S2d). Overall, the data show increased transcription of several inflammasome-related genes in intestinal mucosa of IBD patients with severe disease that correlates with disease activity, suggesting a potential role of inflammasomes in the inflammation of IBD.

### Inflammasome Components Are Differentially Expressed in IECs and LP Cells

**Fig. 3:**
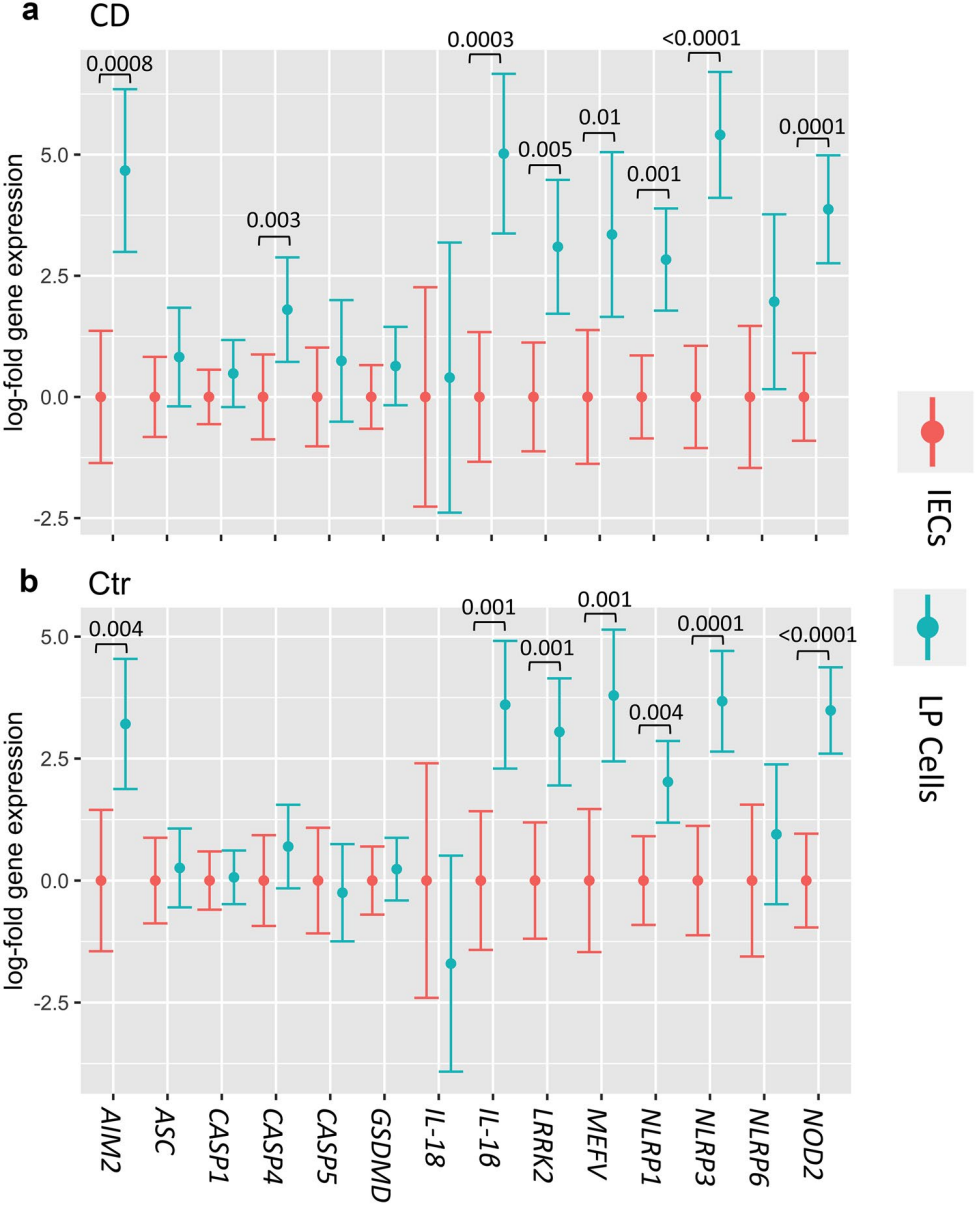
Inflammasome-related gene expression is higher in the LP relative to IECs. Isolated IECs and LP cells were analyzed by RT-PCR using *RPLP0* as the housekeeping gene. Mean log-expression of the indicated genes with 95% confidence intervals measured in IECs (red) and LP cells (blue) from the ileum of (**a**) CD and (**b**) controls is shown. Expression is normalized so mean log-expression in IECs is set to 0 for comparison. Significance was assessed by ANCOVA test and only p values for significant changes (*p* < 0.05) are shown. Gene expression is FACS-adjusted for cell composition (EpCAM^+^, CD45^+^, CD45^−^EpCAM^−^) as gated in Online Resource Fig. S1. Ctr IECs *n* = 7, Ctr LP *n* = 8, CD IECs *n* = 10, CD LP *n* = 9. CD patients were from Cohort 2 (Table 1) and Controls were for CD cohort 2 (Table 2).

Inflammasomes are expressed in IECs and immune cells in the LP, yet the relative contribution of these cellular compartments to expression in CD patients is poorly understood [20]. We thus isolated IECs and LP cells from the ileum of CD patients and controls and examined gene expression. To ensure observed changes reflect the actual IECs versus immune cell composition in the cell fractions, gene expression data was adjusted for the percent of IECs (EpCAM^+^) and immune cells (CD45^+^) determined by flow cytometry for each sample (Online Resource Fig. S1). This data is referred to as FACS-adjusted gene expression. In general, expression of inflammasome genes differed in the cell types irrespective of disease, with several genes being significantly differentially expressed in LP cells relative to IECs (Fig. 3).

Gene-to-gene correlations between inflammasome sensors and effectors revealed that the *MEFV* sensor positively correlated with caspases and *IL-1*β in LP cells from CD patients but not controls (Online Resource Table S2a). Moreover, *MEFV* expression also significantly correlated to caspase genes in IECs from CD patients but not in control IECs (Online Resource Table S2b). *NLRP6*, which is reported to localize in the intestinal epithelium [21], correlated strongly to *CASP1* in CD IECs (Online Resource Table S2b). Overall, the gene expression studies in isolated IECs and LP cells revealed a predominant contribution of LP cells to inflammasome gene expression regardless of disease.

### Inflammasome Expression Increases with the Frequency of Immature Macrophages

We next focused on the contribution of cells purified by sorting to inflammasome gene expression in CD ileum. We thus FACs-sorted IECs (EpCAM^+^CD45^−^), immune cells (CD45^+^), non-epithelial/non-immune cells (EpCAM^−^CD45^−^; called stromal cells), and total MNPs (CD14^+^HLA-DR^+^) for transcriptional analyses (Online Resource Fig. S3a). It is well documented that MNPs, particularly immature macrophages, greatly increase in inflamed intestinal tissue of CD patients [3, 4, 12]. In contrast, MNPs including immature macrophages are very scarce in non-inflamed control tissue [3, 4, 12], which precluded sorting enough of these cells from controls for RT-PCR analyses. Indeed, even with the influx of immature macrophages in inflamed tissue, their number after sorting was still relatively low per individual. This necessitated pooling mRNA of a given cell type from CD patients to give sufficient material for RT-PCR analysis. Gene expression data of sorted cells showed that the sensors *NLRP3* and *MEFV,* as well as *IL-1β*, were highly expressed by MNPs (Online Resource Fig. S3b).

**Fig. 4:**
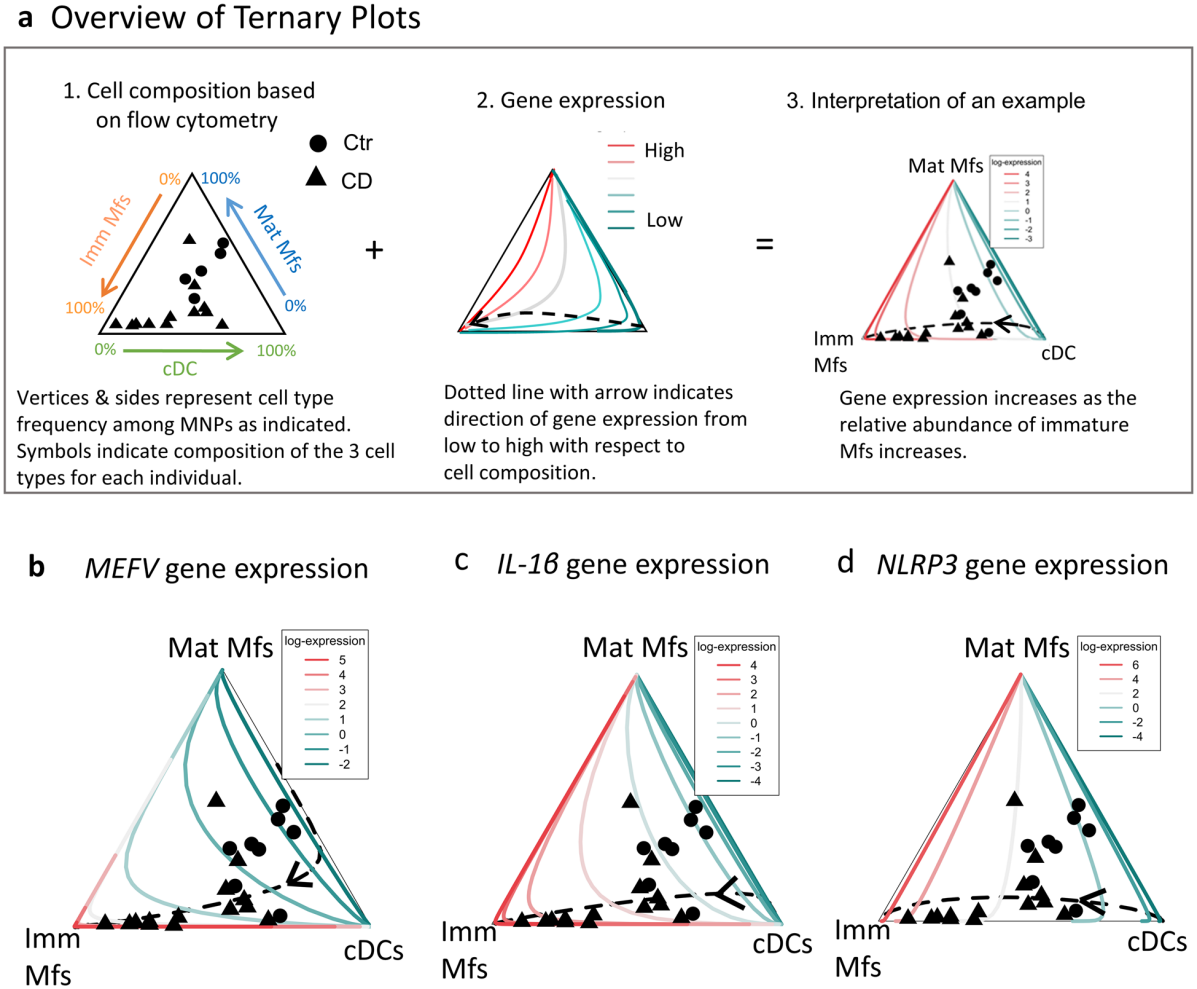
*MEFV* and *NLRP3* expression increase with the relative abundance of immature Mfs. Gene expression (log_2_) of *MEFV*, *IL-1*β and *NLRP3* in LP cells from an individual adjusted for cell composition in the same LP cells determined by flow cytometry of immature Mfs (Imm Mf; CD14^+^CD11c^+^), mature Mfs (Mat Mfs; CD14^+^CD11c^−^), and cDCs (CD14^−^CD11c^−^), gated as in Online Resource Fig. S1, are shown. Ternary plots are shown where each diagram integrates the frequency of a specific cell type (measured by flow cytometry), gene expression (measured by RT-PCR) and scatter plot of the individual samples. (**a**) An example of how to interpret the diagrams is shown, with an overview as follows. (a1) Cell composition is modeled based on flow cytometry analysis using Mf and cDC surface markers. Vertices represent the indicated cell type frequency among total MNPs ranging from 100 to 0% along each side. The symbols represent CD (triangles) or controls (circles) and indicate where each sample is located with respect to the composition of the three cell types. (a2) Illustrates gene expression from lowest to highest. The dashed line indicates expression from lowest (green) to highest (red), in the direction of the arrow, with respect to the cell composition. (a3) Interpretation of an example. (**b**–**d**) Gene expression of *MEFV*, *NLRP3,* and *IL-1*β with respect to the three cell types. Ternary plots for the indicated genes are plotted. Ctr *n* = 8, CD *n* = 14. CD patients were from Cohort 2 (Table 1) and Controls were for CD cohort 2 (Table 2).

We next sought to assess the contribution of specific MNP populations to inflammasome gene expression. Given the technical difficulty of sorting enough of each MNP population per individual to analyze multiple genes, we used an alternative approach to assess gene expression in specific MNP populations. We thus incorporated the FACS-frequency of a given MNP population in LP cells of an individual and gene expression data in the same LP cells using compositional analysis. This approach allowed us to extend gene expression observations into immature Mf, mature Mf, and cDC populations [3, 12] (defined in Online Resource Fig. S1b). Figure 4a shows an overview of this strategy. Indeed, expression of *MEFV* and *IL-1*β increased as the relative abundance of immature Mfs increased (Fig. 4b–c). This is shown in Fig. 4b–c by the arrow direction going toward the Imm Mf vertex where there is also high gene expression (red color) at the Imm Mf vertex. Expression of *NLRP3* increased as the relative abundance of immature and mature Mfs increased, with immature Mfs having a greater impact (Fig. 4d). This is shown in Fig. 4b–c by the arrow direction going toward the Imm Mf vertex with high gene expression (red color) being a flat line along the side between Imm Mfs and Mat Mfs. Overall, these data show cell type-specific expression of inflammasome genes among three populations of MNPs. Among these three populations, immature Mfs, which greatly increase in inflamed tissue of CD patients [3, 12], have the greatest impact on *MEFV*, *IL-1β* and *NLRP3* expression.

### Serum from CD Patients Has a Distinct Protein Profile

**Fig. 5:**
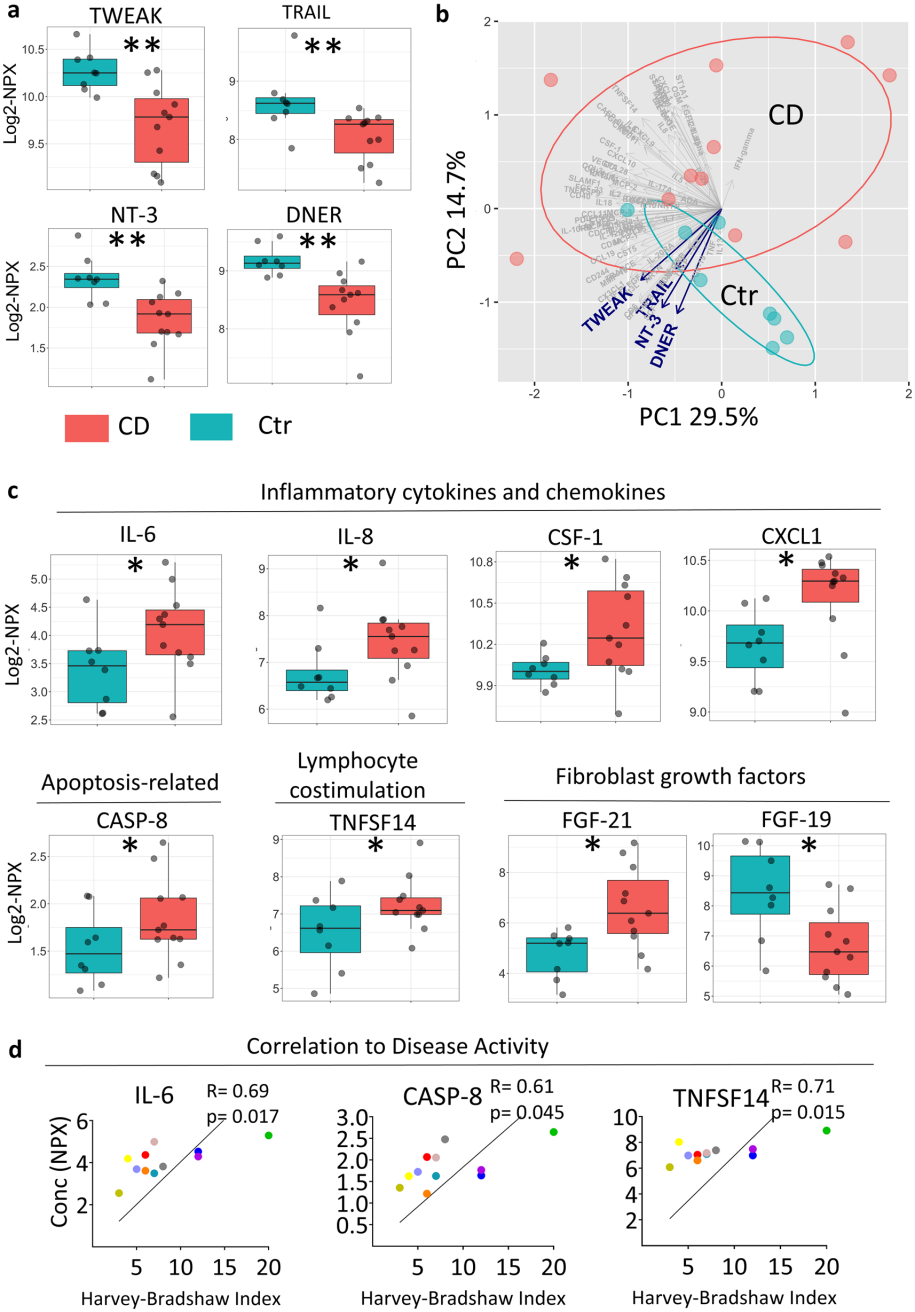
CD patients have a distinct serum protein profile compared to age- and sex-matched controls. Serum proteins were analyzed by proximity extension assay using the 92 protein Inflammation Panel (Olink) and are presented in the arbitrary unit normalized protein expression (NPX). Differentially expressed serum proteins of CD patients compared to healthy controls were calculated with adjustment for age- and sex-matched strata as random effects. (**a**) Boxplots of proteins with the lowest false discovery rate (FDR < 5%) are shown. (**b**) Principal component analysis (PCA) plot with respect to all proteins is shown with the x- and y-axis indicating the % explaining the variation of PC1 and PC2. (**c**) Boxplots of eight differentially expressed proteins (FDR < 10%) divided by function are shown. (**d**) Linear regression plots showing Pearson correlation of the indicated serum proteins with disease activity (Harvey Bradshaw Index) are shown. Each dot represents an individual. Ctr *n* = 8, CD *n* = 11. CD patients were from cohort 2 (Table 1) and Controls were for CD cohort 2 (Table 2).

We next characterized the serum inflammatory profile of CD patients and healthy age-/sex-matched controls. This revealed 16 proteins that were significantly changed in serum of CD patients (Table 3). Notably, the four proteins with the lowest false discovery rate (FDR < 0.05), TWEAK, TRAIL, NT-3, and DNER (blue in Table 3), were reduced by at least 30% in CD patients relative to controls (Fig. 5a). Indeed, there was separation between CD patients and controls along the axis defined by these four proteins in the first two principal components of the protein dataset (Fig. 5b). Other significantly differentially expressed serum proteins in CD patients compared to controls include cytokines, chemokines, and IBD-related markers (Fig. 5c). Interestingly, the NLRP3-related apoptosis protein CASP-8 was among the differentially expressed proteins in serum from CD patients (Fig. 5c, Table 3), and *CASP8* gene expression was significantly upregulated in the ileum of CD patients (Fig. 2a). The significantly differentially expressed serum protein TNFSF14 (Table 3) correlated with age (*R* = 0.53; *p* = 0.02), supporting the importance to use age-matched controls. Moreover, three of the proteins differentially expressed in serum from CD patients significantly correlated with disease activity (Fig. 5d), suggesting they may have potential as biomarkers. Overall, the serum of CD surgical patients had increased inflammatory proteins including cytokines, decreased TNF-superfamily members TWEAK and TRAIL as well as reduced levels of the newly identified NT-3 and DNER.

**Table 3:** Differentially Expressed Inflammatory Proteins in the Serum of CD Patients

Protein	Fold change	*p* value	FDR	Differentially expressed in CD	Below LOD (%)
FGF-21	3.58	0.011	0.057	Upregulated	0
ST1A1	2.95	0.018	0.058	Upregulated	9.52%
TNFSF14	1.68	0.037	0.081	Upregulated	0
IL-6	1.65	0.012	0.057	Upregulated	0
IL-8	1.60	0.049	0.083	Upregulated	0
VEGFA	1.59	0.039	0.081	Upregulated	0
CXCL1	1.39	0.028	0.077	Upregulated	0
CASP-8	1.24	0.051	0.083	Upregulated	0
CSF-1	1.22	0.036	0.081	Upregulated	0
GDNF	0.84	0.029	0.077	Downregulated	4.76%
uPA	0.71	0.015	0.058	Downregulated	0
NT-3	0.70	0.004	0.04	Downregulated	4.76%
TWEAK	0.67	0.006	0.042	Downregulated	0
TRAIL	0.66	0.001	0.036	Downregulated	0
DNER	0.62	0.004	0.04	Downregulated	0
FGF-19	0.30	0.017	0.058	Downregulated	0

Proteins significantly differentially expressed in the serum of CD patients compared to healthy age- and sex-matched controls (estimated normalized expression, expressed as log_2_NPX) are shown, ordered from highest to lowest fold change. Fold difference is expressed as 2^estimate. All proteins shown have a false discovery rate (FDR) of <10% and the four with the lowest false discovery rate (FDR < 5%; NT-3, TWEAK, TRAIL and DNER) are shown Fig. 5a,b. CD patients are CD cohort 2 n=11. Controls are those for CD cohort 2 (Table 2), n=8

#### Serum Proteins Correlate with Ileal Inflammasome Gene Expression in CD in Paired Sample Analysis

A goal of this study was to correlate the systemic (serum) inflammatory profile with intestinal gene expression in the same CD patient using paired samples from the same individual. Such a link may facilitate screening for altered intestinal inflammasome expression using a readily available clinical sample (blood) to, for example, develop biomarkers for treatments targeting IL-1 family cytokines [2]. We thus correlated the serum protein profile of CD patients with ileal gene expression of the inflammasome sensors *MEFV*, *NLRP3,* and *NLRP6* in paired sample analysis. First, FACS-adjusted *MEFV* expression in LP cells negatively correlated with several serum proteins (Online Resource Table S3a). Second, LP expression of *NLRP3* correlated positively with serum CXCL11 and negatively with IL-6 in paired sample analyses of CD patients. Third, *NLRP6* expression in LP correlated positively with IL-18 and the corresponding receptor IL-18R1 and with the chemokines MCP-1 and MCP-2 (Online Resource Table S3a). Moreover, *NLRP6* expression in IECs also positively correlated with several inflammatory serum proteins including TRAIL, which is differentially expressed in CD, as well as IL-18 and IL-17C (Table 3, Online Resource Table S3b).

**Fig. 6:**
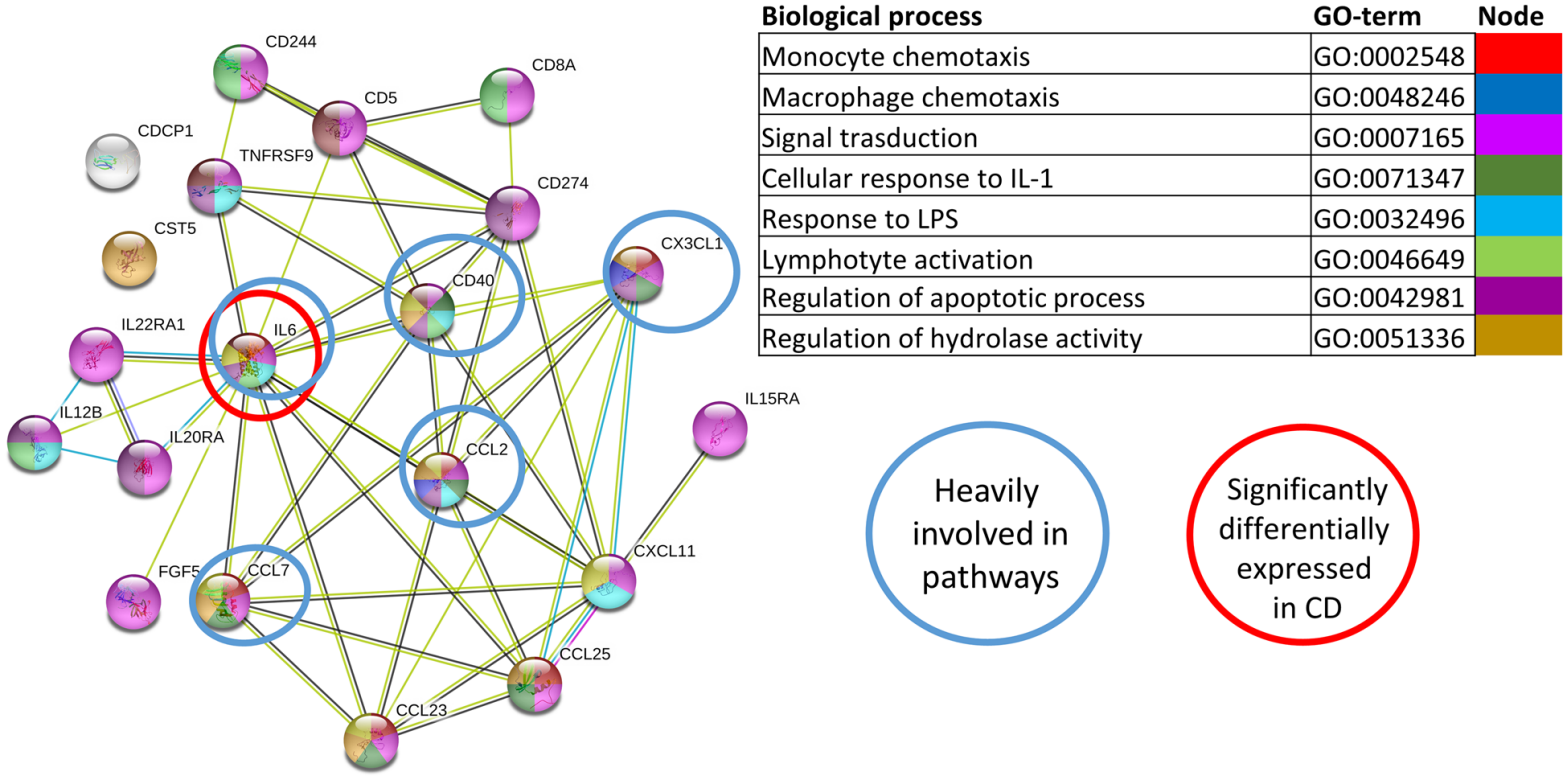
Correlation of MEFV and NLRP3 inflammasome-related gene expression in LP with serum proteins using paired serum and tissue samples from CD patients. FACS-adjusted gene expression of inflammasome genes in LP of CD ileum was correlated with paired serum proteins from the same patients. The STRING network, according to GO-term, of the 20 serum proteins that correlate with MEFV and NLRP3 inflammasome-related genes in the LP (*MEFV*, *NLRP3*, *CASP1*, *IL-1*β, *IL-18* and *ASC*) is shown. Pathways were selected based on their implication with inflammasomes, macrophages, and IBD. CD *n* = 6. CD patients were from cohort 2 (Table 1) and paired tissue and blood samples from these patients were used.

Protein association networks indicated that serum proteins correlating with expression of *MEFV-* and *NLRP3*-associated genes in LP cells from CD patients in paired samples (Online Resource Table S3a and data not shown) were involved in inflammatory processes (Fig. 6). In particular, IL-6 was differentially expressed in serum from CD patients (Table 3, Fig. 5c), negatively correlated with *NLRP3* expression in CD LP cells (Online Resource Table S3a), and is heavily involved in inflammatory pathways (Fig. 6). In summary, *NLPR3* expression in LP cells from CD patients negatively correlates with the differentially expressed serum protein IL-6, while *NLRP6* expression IECs strongly correlate positively with IL-18 and with the differentially expressed protein TRAIL.

### IL-1β Secretion in CD Ileum Is Attributed to Immature Macrophages

**Fig. 7:**
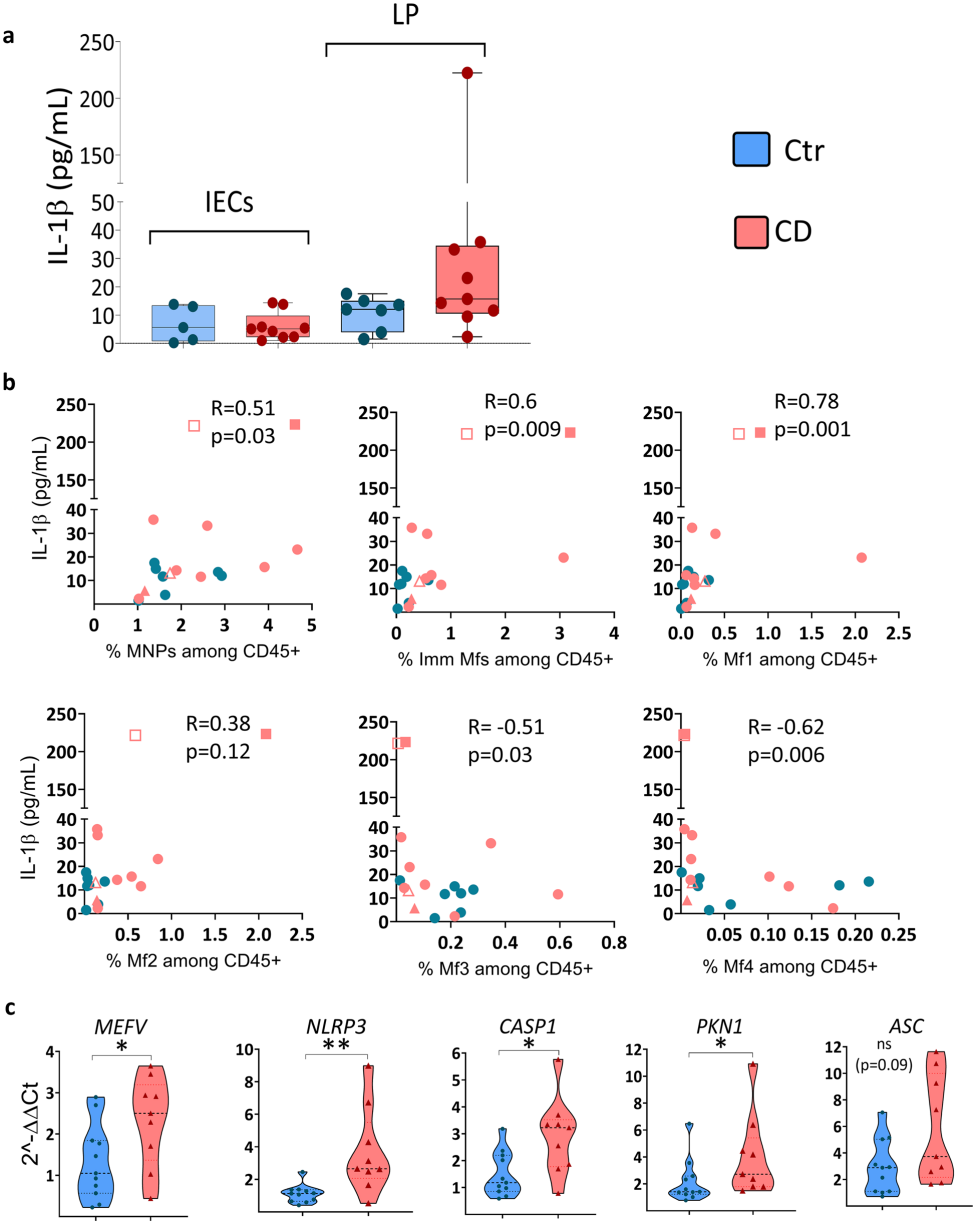
IL-1β release from LP cells correlates with immature macrophage frequency. (**a**) Spontaneous IL-1β release was measured by ELISA from LP-conditioned media (LP-CM) and IEC-CM cultured ex vivo for 22 h. (**b**) Spearman correlation between IL-1β in LP-CM and the frequency of the indicated Mf subsets among total immune cells (CD45^+^ cells) (Online Resource Fig. S1) in paired samples from the same control individuals (***n*** = 7) and in paired samples from the same CD patients (n = 9) is shown. For CD, two of the patients had biological duplicates shown as open and filled squares for one patient and open and filled triangles for the other. (**c**) Gene expression of the indicated genes in PBMCs from CD patients compared to controls is shown. Violin plots showing all points are ploted for controls (*n* = 11) and CD patients (*n* = 9). Genes were analyzed by RT-PCR using *RPLP0* as the housekeeping gene and data are shown as relative fold change to control (Ctr) using the 2^−ΔΔCT^ method. Significance was assessed by Mann–Whitney U test, *p < 0.05, **p < 0.005, ***p < 0.0005. CD patients were from cohort 2 (Table 1) and Controls were for CD cohort 2 (Table 2).

The data suggest that expression of *MEFV* and *NLRP3* is high in immature LP Mfs, which greatly increased in inflamed tissue of CD patients [3, 12]. Indeed, IL-1β was spontaneously released from LP cells of CD patients (Fig. 7a). Secreted IL-1β levels also positively correlated with immature Mf frequency, particularly the Mf1 subset, which is the most immature and is related to circulating monocytes (Fig. 7b) [3, 22]. Furthermore, IL-1β negatively correlated with the frequency of mature Mf subsets (Mf3, Mf4) (Fig. 7b), which are less reactive to inflammatory stimuli [22].

Given the strong correlation of monocyte-related, immature Mfs and IL-1β production (Fig. 7b) and the altered serum protein profile of CD patients (Fig. 5), we asked if inflammasome genes are already increased in PBMCs from patients with CD. Indeed, *MEFV*, *NLRP3*, and *CASP1* expression was significantly increased, while *ASC* tended to increase, in PBMCs from CD patients relative to those from controls (Fig. 7c). An effector thought to be involved in the *MEFV*-associated RhoA signaling pathway, PKN1, was also increased in PBMCs from CD patients (Fig. 7c). Thus, IL-1β release as an indication of inflammasome activation in LP cells of CD patients correlates with immature Mf frequency, particularly the Mf1 subset [22] that increases significantly in inflamed CD intestine [3, 22]. Moreover, transcription of MEFV and NLRP3 inflammasome-related genes is increased in PBMCs of CD patients.

## Discussion

In an approach using mucosal tissue, IEC and LP cell fractions, FACS-sorted cells and PBMCs, we show that *NLRP3* and *MEFV* inflammasomes are differentially expressed in CD. In particular, transcription of the sensors *NLRP3* and *MEFV*, the effector *CASP1,* and its product IL-1β are increased in the ileum of CD patients, with immature Mfs having the greatest impact on increased expression. Indeed, Mfs are greatly increased in inflamed intestine of IBD, particularly immature Mfs [3, 12]. They have an inflammatory phenotype [4, 11, 12], derive from circulating CD14^+^ monocytes, and mature into homeostatic Mfs [3, 22, 23]. The influx of immature macrophages with an inflammatory phenotype to inflamed intestine [3, 4, 11, 12] likely accounts for increased expression of inflammasome genes at the whole tissue level. Consistent with the monocyte origin of immature Mfs [3, 22], expression of several inflammasome genes was already increased in PBMCs of CD patients relative to controls. Indeed, similar to our findings, *NLRP3* in SARS-Cov-2-infected patients is activated already in PBMCs, and its expression correlates with serum markers of COVID-19 severity [24]. Thus, expression of inflammasome genes in PBMCs and correlation to serum protein profiles may be a valuable way to assess disease severity using readily accessible patient samples.

The signals and disease-specific physiological ligands guiding inflammasome activation in circulating cells remain to be defined. However, serum contains microbial components and cellular damage molecules that can influence inflammasome activation [25, 26]. Consistently, our String network analysis revealed that inflammatory proteins in serum from CD patients that correlate with ileal inflammasome gene expression are involved in pathways including the response to LPS and TLR signaling. Our finding that IL-1β released from LP cells of CD patients is associated with immature Mfs, together with increased inflammasome gene expression in these cells and their circulating precursors, support a role for MEFV and NLRP3 inflammasomes in immature Mfs in driving CD ileitis.

Studies of NLRP3 suggest a role in IBD [7], and our data provide further support. In addition to increased *NLRP3* expression itself, we reveal increased expression of several effectors downstream of this sensor [27–30]. This includes *CASP8*, whose transcription increased in CD ileum, while CASP-8 protein was elevated in serum of CD patients and correlated with disease activity. CASP-8 is an effector of NLRP3 signaling [31] via FADD-CASP-8 complexes that function as a scaffold to support cellular signaling via TRAIL-R [32]. In addition to NLRP3, the MEFV/Pyrin inflammasome has also been implicated in IBD pathogenesis [33]. Indeed, we found that *MEFV* expression strongly correlates with *CASP-1* and *IL-1*β in LP cells from CD patients, with inflammatory serum proteins in CD patients, and with disease activity in both CD and UC.

We aimed to determine if mucosal inflammation, as distinguished by altered gene expression in mucosal tissue, was reflected in the serum of the same CD patient. This is valuable given the ease of obtaining serum in a clinical setting and the need to identify new biomarkers for parameters such as diagnosis, disease progression, and response to treatment. The surgical CD patients in this study had a distinct serum protein profile compared to age- and sex-matched controls. Indeed, a subset of the elevated inflammatory proteins, IL-6, IL-8, CASP-8, and CXCL1, was also found among differently expressed serum proteins in random (non-surgical) CD cohorts using the same method as here (proximity extension assay) [34–36]. Moreover, we further show that two of these proteins, IL-6 and CASP-8, positively correlated with disease severity in our surgical CD cohort. This suggests that identifying serum protein signatures in readily available clinical samples, such as in serum of suspected or confirmed CD [34–36] and patients with complications necessitating surgery as shown here, may lead to biomarkers for diverse clinical applications such as diagnosis, prognosis, or disease severity.

The serum of our CD patients also had significantly reduced FGF-19, NT-3 and DNER as well as reduced levels of TNF-superfamily members TRAIL (TNFSF10) and TWEAK (TNFSF12). Among these proteins, FGF-19, NT-3 and DNER were also significantly reduced in the serum of non-surgical cohorts [34, 36], suggesting similarity in CD patients despite disease complications necessitating surgery. These data warrant further study given that NT-3 promotes intestinal mast cell survival [37] and DNER regulates IFNγ secretion in recruited Mfs during pulmonary inflammation [38]. Furthermore, TRAIL and TWEAK are involved in apoptosis in the intestinal mucosa. While TRAIL is downregulated in enterocytes of IBD patients, it is upregulated in mononuclear cells in areas of active mucosal inflammation [39]. During inflammation, TRAIL-expressing mononuclear cells become a potent inducer of apoptosis in IECs [40, 41], which may underlie the positive correlation of TRAIL with NLRP6 in IECs. The TWEAK receptor Fn14 is found on T cells, Mfs, and DCs in intestinal mucosa [41], and epithelial cells upon encountering TWEAK are sensitized to TNF-induced death [40]. Indeed, TWEAK alteration seems to contribute to CD pathogenesis [42], but the few reports on circulating levels are inconclusive, possibly due to cohort differences [43].

Interestingly, some proteins in the serum of CD patients negatively correlated with expression of genes related to NLRP3 and MEFV inflammasomes in LP cells in paired sample analysis. This could reflect increased recruitment of circulating immune cells to the LP, particularly MNPs [4, 11, 12], where inflammasome gene expression is potently induced by the local (intestinal) milieu. Local increases in inflammasome gene expression may not necessarily be reflected in systemic (serum) increases of proteins. Indeed, serum proteins can be produced by, and act on, a variety of cell types. Our data showing a negative correlation of LP *MEFV* expression with serum proteins that influence lymphocytes, including CCL25, CD5, and CD8A, supports this notion. Similarly, although IL-6 is upregulated in CD serum, its concentration negatively correlated with LP *NLRP3* expression. Thus, serum IL-6 does not reflect increased *NLRP3* expression by LP cells, particularly immature macrophages that infiltrate inflamed intestine of CD patients. Although IL-6 is involved in driving IBD inflammation [2, 6] and may be involved in inflammasome pathways, no consensus is apparent whether IL-6 regulation is inflammasome-related or a consequence of inflammatory processes involving macrophage exacerbation [6, 44, 45].

In contrast to *MEFV* and *NLRP3*, *NLRP6* expression in LP cells from CD patients correlated positively with serum proteins in the same individuals. In particular, we show for the first time in CD that intestinal *NLRP6* expression positively correlates with serum IL-18 and IL-18R1 in paired sample analysis, which was only previously examined in mice [46]. The strongest correlation was for *NLRP6* expression in IECs with serum IL-18. In addition, paired sample analysis also revealed that IEC expression of *NLRP6*, as well as *CASP1* (data not shown), positively correlated with TRAIL, which is strongly differentially expressed in CD patient serum. The positive correlation of serum TRAIL with *NLRP6* and *CASP1* expression in IECs suggests a link between TRAIL and IEC inflammasomes, possibly via apoptotic processes that merge on activation of caspases.

Ideal control intestinal tissue for studies like ours is from healthy individuals. However, for ethical reasons, such tissue is not available in sufficient amounts (from surgical rection) to perform multiple parallel analyses as we have done here. Our control tissue was thus mostly from patients undergoing surgery for CRC, and data obtained with this tissue must be considered with this caveat. Despite this limitation, macroscopically normal tissue as far away from tumor as possible (> 10 cm), at the surgical resection margin, was always used. Moreover, the CRC patients were not of advanced stage (no stage-4 patients were included). For our studies with serum, we chose to use healthy blood donors as controls. This facilitated matching age and sex for each CD patient, as we indeed found some age-related differences in serum proteins as stated above.

Patients with CD are highly heterogeneous, and means to reliably stratify into subgroups to increase treatment success is highly desirable [47]. Characterization of inflammasome-related parameters, as investigated here, may facilitate CD stratification to improve therapy success. For example, inflammasome sensors or IL-1β expression in intestinal biopsies could be explored, particularly in combination with altered serum proteins, in samples taken during clinical visits. Moreover, serum IL-18 or TRAIL, which correlate with mucosal *NLRP6*, could be indicators of inflammasome activation and suggest therapies such as blocking the IL-1 family [2, 6]. Indeed, therapy using NLRP3 inhibitors is currently being explored in a phase 1 clinical trial (ClinicalTrials.gov: NCT04015076).

In conclusion, our study provides further evidence for the importance of NLRP3 and MEFV inflammasomes in CD pathogenesis. This is supported by gene expression data from inflamed mucosa of CD patients and from several cell types (IECs, LP cells, FACS sorted cells and PBMCs). We also show that some serum proteins of CD patients correlate to inflammasome expression at the mucosal level. Finally, as *NLRP3* and *MEFV* were highly expressed by pro-inflammatory Mfs in the mucosa as well as among PBMCs, we suggest that monocytes recruited to the inflammatory site may be pre-programmed for altered inflammasome function when located to the inflamed mucosa. Overall, further research of the role of inflammasomes in CD patients can provide deeper knowledge to design better treatment targets for patients with CD or identify patient subgroups that could benefit from specific treatments such as IL-1β blockers.

## Supplementary Information

Below is the link to the electronic supplementary material.

Online Resource Fig. S1 Flow cytometry gating strategy. The gatings used to determine the frequency of the indicated cell populations among IECs (**a**) and LP cells (**b**) are shown using an ileal sample from a CD patient. (**a**) and the upper left plot of (**b**) show gating for the EpCAM+, CD45+ and EpCAM-CD45- populations used for FACS-adjusted gene expression analysis. The additional gates shown in (**b**) define the total MNP population (CD14+HLA-DR+), cDCs, total immature Mfs and subsets thereof (Mf1 and Mf2) as well as total mature Mfs and subsets thereof (Mf3 and Mf4). CD patients were from Cohort 2 (Table 1) (TIF 5567 KB)

Online Resource Fig. S2 Gene expression of inflammasome components in UC colonic mucosa. Colonic mucosa from UC patients (UC cohort in Table 1) was analyzed by RT-PCR. Gene expression was normalized to the housekeeping gene RPLP0. (**a**-**c**) Scatter plots of genes with significant differences in gene expression in UC compared to controls (same colonic controls as used in Fig. 1). Data are shown as relative fold change to control (Ctr) using the 2^-ΔΔCT^ method. Bar height indicates the median. Significance was assessed by Mann-Whitney U test, *p<0.05, **p<0.005, ***p<0.0005. (**d**) Linear regression plots show Pearson correlation for expression of the indicated genes with disease score (Full Mayo Score). Scatter plots were plotted using 2^-ΔCT^ and each dot represents a patient. Ctr n=10, UC n=9. UC patients are shown in Table 1 and Controls were for CD cohort 1 and UC cohort (Table 2) (TIF 2362 KB)

Online Resource Fig. S3 MEFV and NLRP3 inflammasome-related genes are highly expressed by MNPs. (**a**) Cells from CD ileitis patients were FACS-sorted into four cell populations [epithelial cells (EpCAM^+^), total immune cells (CD45^+^), non-epithelial/non-immune cells (EpCAM^-^CD45^-^, named stromal cells) and total MNPs (CD14^+^HLA-DR^+^)] for RT-PCR analysis. (**b**) Expression of the indicated genes in the four sorted cell populations is shown as 2^-ΔCT^. Before RNA extraction, the same sorted cell population from ≥ 4 CD patients was pooled to obtain enough material for RT-PCR. Thus, each bar represents expression of the sample pool. CD patients were from Cohort 2 (Table 1) (TIF 2188 KB)

Online Resource Table S1 Primers used for gene expression analyses. (**a**) Primers used for gene expression analysis are shown by category to indicate the function/components of each gene within the inflammasome. (**b**) List of the genes added to the commercial NanoString nCounter Fibrosis panel. CD patients were from Cohort 2 (Table 1) and Controls were for CD cohort 2 (Table 2) (XLSX 15 KB)

Online Resource Table S2 Gene-to-gene correlations of inflammasome sensor and downstream effector gene expression of LP and IECs cells. Correlations between gene expression of inflammasome sensors (*MEFV*, *NLRP3*, *NLRP6*, *AIM2* and *NLRP1*) and effectors, indicated in the respective columns, for (**a**) LP and (**b**) IECs from controls and CD ileum are shown. Gene expression is FACS-adjusted for cell composition of EpCAM^+^, CD45^+^, CD45^-^EpCAM^-^ as gated in Online Resource Fig. S1. Significant correlations (p<0.05) are highlighted in yellow. Ctr IECs n=7, CD IECs n=10, Ctr LP n=8, CD LP n=9. CD patients were from Cohort 2 (Table 1) and Controls were for CD cohort 2 (Table 2) (XLSX 14 KB)

Online Resource Table S3 Significant correlations of MEFV, NLRP3 and NLRP6 inflammasome-related gene expression to serum proteins in paired CD ileitis samples. Significant correlations (p<0.05) between expression of genes related the MEFV, NLRP3 and NLRP6 inflammasomes with serum proteins detected in CD patients are shown. Genes in (**a**) LP cells and (**b**) IECs from CD patients using paired mucosa-serum samples are shown. Gene expression is FACS-adjusted for cell composition (EpCAM^+^, CD45^+^, CD45-EpCAM^-^; Online Resource Fig. S1). For gene expression analysis, n=6 for LP and n=7 for IECs. For serum proteins, n=7 CD. CD patients were from Cohort 2 (Table 1) (XLSX 14 KB)

## Data Availability

The datasets generated during and/or analyzed during the current study, and the materials used, are available from the corresponding author on reasonable request.
