# Supplementary figures and images for: MEFV and NLRP3 Inflammasome Expression Is Attributed to Immature Macrophages and Correlates with Serum Inflammatory Proteins in Crohn´s Disease Patients
Source: Inflammation. 2022 Feb 21;45(4):1631–50. doi: 10.1007/s10753-022-01647-8 (PMC8860375; doi:10.1007/s10753-022-01647-8)

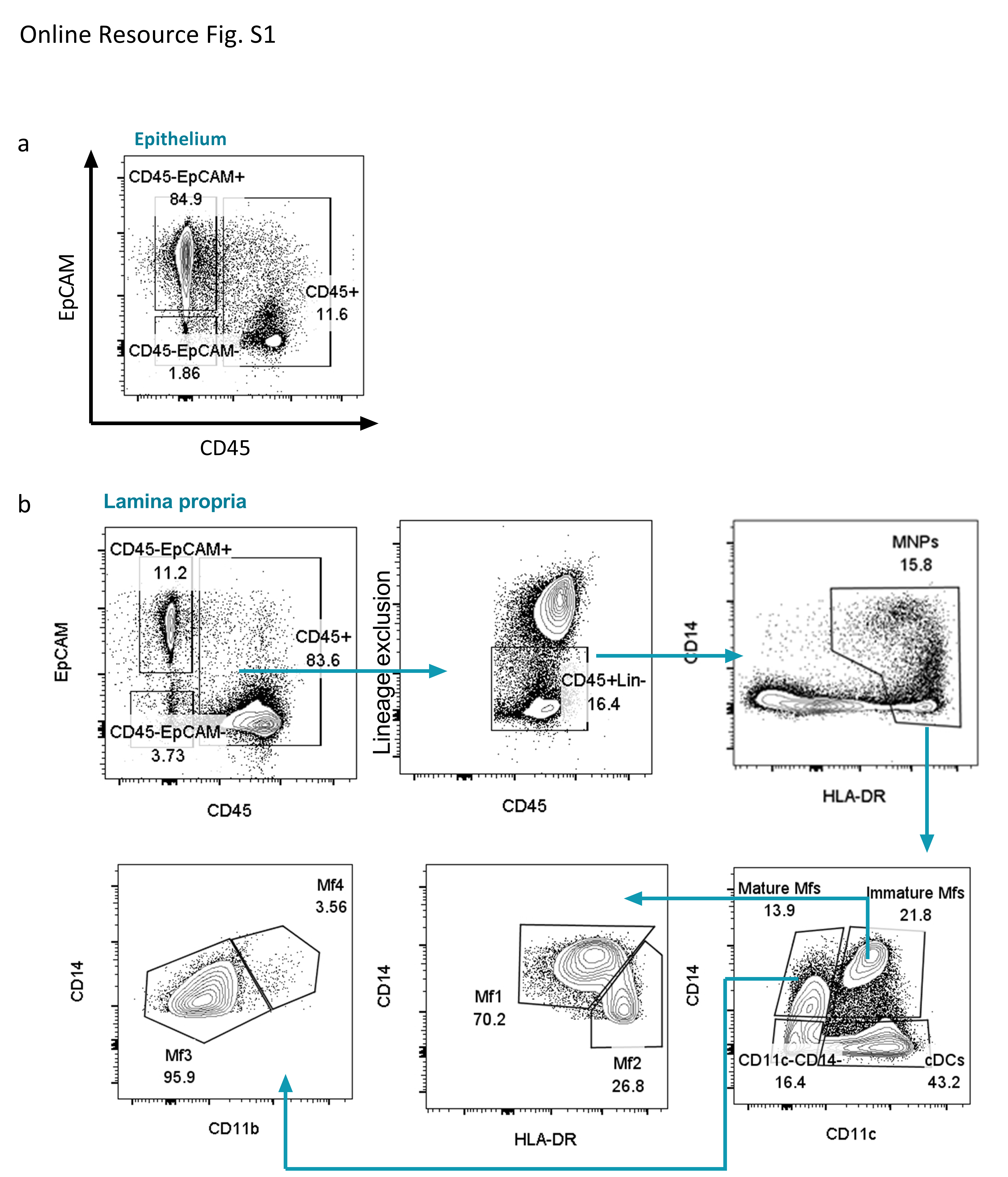

Supplement: Supplementary file 1 — Online Resource Fig. S1 Flow cytometry gating strategy. The gatings used to determine the frequency of the indicated cell populations among IECs (a) and LP cells (b) are shown using an ileal sample from a CD patient. (a) and the upper left plot of (b) show gating for the EpCAM+, CD45+ and EpCAM-CD45- populations used for FACS-adjusted gene expression analysis. The additional gates shown in (b) define the total MNP population (CD14+HLA-DR+), cDCs, total immature Mfs and subsets thereof (Mf1 and Mf2) as well as total mature Mfs and subsets thereof (Mf3 and Mf4). CD patients were from Cohort 2 (Table 1) (TIF 5567 KB) [file 10753_2022_1647_MOESM1_ESM.tif]

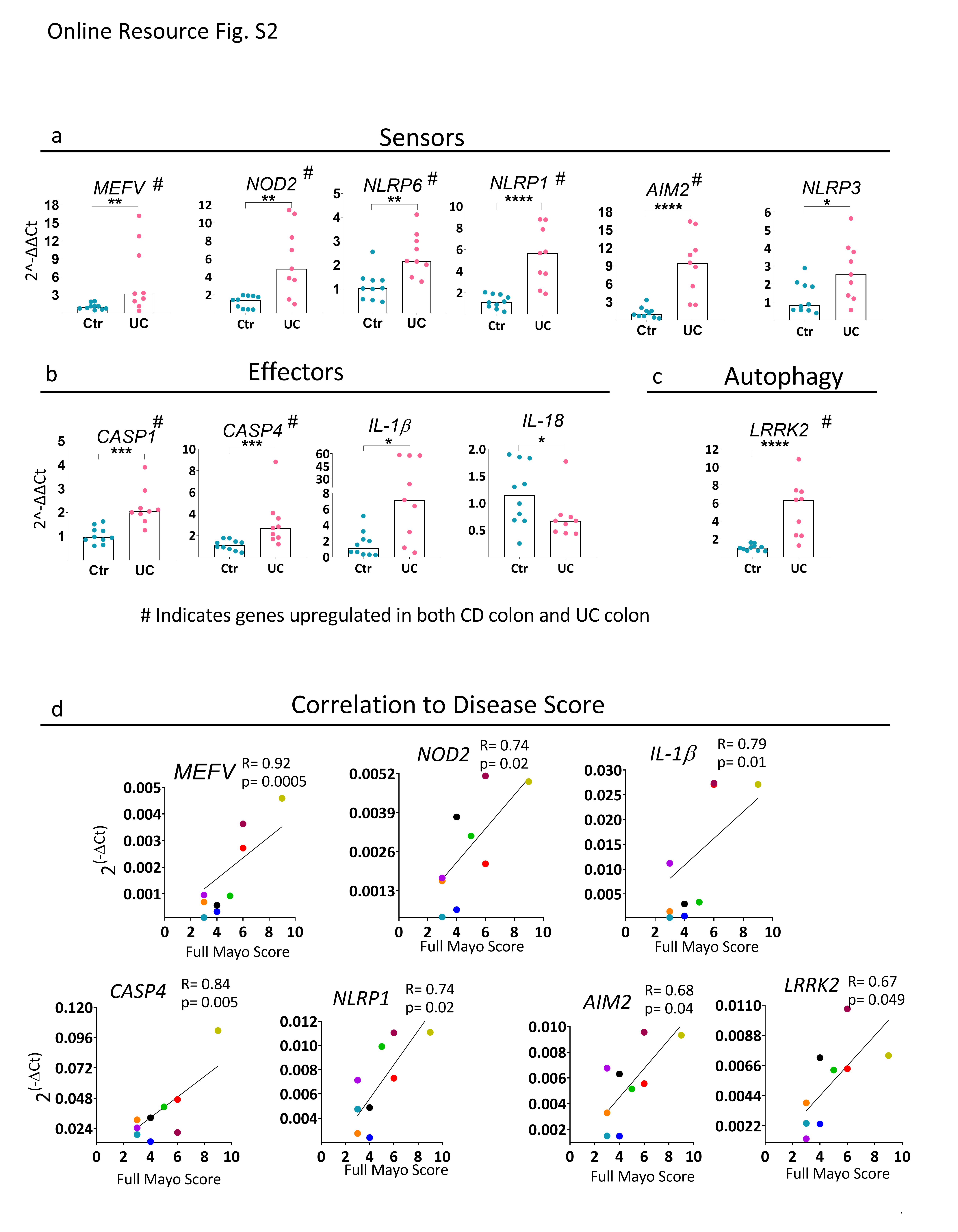

Supplement: Supplementary file 2 — Online Resource Fig. S2 Gene expression of inflammasome components in UC colonic mucosa. Colonic mucosa from UC patients (UC cohort in Table 1) was analyzed by RT-PCR. Gene expression was normalized to the housekeeping gene RPLP0. (a-c) Scatter plots of genes with significant differences in gene expression in UC compared to controls (same colonic controls as used in Fig. 1). Data are shown as relative fold change to control (Ctr) using the 2-ΔΔCT method. Bar height indicates the median. Significance was assessed by Mann-Whitney U test, *p<0.05, **p<0.005, ***p<0.0005. (d) Linear regression plots show Pearson correlation for expression of the indicated genes with disease score (Full Mayo Score). Scatter plots were plotted using 2-ΔCT and each dot represents a patient. Ctr n=10, UC n=9. UC patients are shown in Table 1 and Controls were for CD cohort 1 and UC cohort (Table 2) (TIF 2362 KB) [file 10753_2022_1647_MOESM2_ESM.tif]

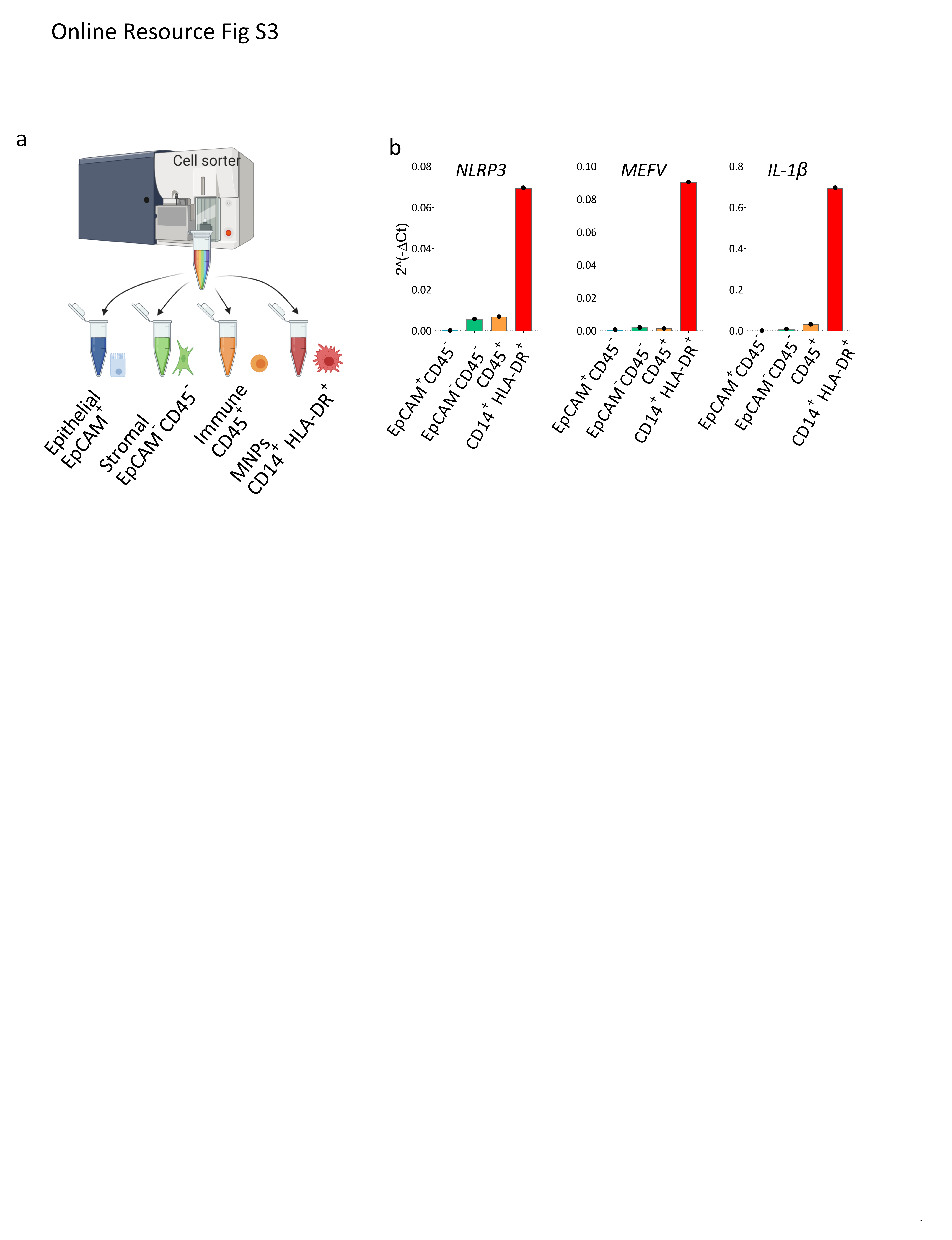

Supplement: Supplementary file 3 — Online Resource Fig. S3 MEFV and NLRP3 inflammasome-related genes are highly expressed by MNPs. (a) Cells from CD ileitis patients were FACS-sorted into four cell populations [epithelial cells (EpCAM+), total immune cells (CD45+), non-epithelial/non-immune cells (EpCAM-CD45-, named stromal cells) and total MNPs (CD14+HLA-DR+)] for RT-PCR analysis. (b) Expression of the indicated genes in the four sorted cell populations is shown as 2-ΔCT. Before RNA extraction, the same sorted cell population from ≥ 4 CD patients was pooled to obtain enough material for RT-PCR. Thus, each bar represents expression of the sample pool. CD patients were from Cohort 2 (Table 1) (TIF 2188 KB) [file 10753_2022_1647_MOESM3_ESM.tif]
